# Process Optimization and Aroma Composition Study of Wine from Buddha’s Hand, Citron, Galangal

**DOI:** 10.3390/foods14111936

**Published:** 2025-05-29

**Authors:** Minglong Li, Kaiyue Zheng, Ling Li, Yuxin He

**Affiliations:** 1 School of Food and Bioengineering, Xihua University, Chengdu 610039, China; cyylml123456@126.com (M.L.); 17767916603@163.com (K.Z.); 2 School of Comprehensive Health Management, Xihua University, Chengdu 610039, China

**Keywords:** Buddha’s hand, citron, galangal, fermentation process optimization, compositional analysis

## Abstract

Buddha’s hand, citron, and galangal are widely used in the food and pharmaceutical fields. In this study, the type of yeast, *Saccharomyces cerevisiae* addition, pH, extract content, and fermentation temperature were used as the investigating factors, and the sensory scores, alcohol, reducing sugar, total phenol content, and total flavonoid content of fermented wine were used as the investigating indexes. The fermentation conditions of fermented wine were optimized using the Box-Behnken design-response surface method combined with the entropy weighting method; HS-SPME-GC-MS performed the compositional analyses. The results demonstrated that the wine made from Buddha’s hand, citron, and galangal exhibited a unique aroma, seamlessly blending herbal and fruity notes. This product contained components at relatively high concentrations that are known to possess potential anti-inflammatory, antioxidant, and sleep-promoting properties, among others. These findings provide a valuable foundation for the further development and resource utilization of this innovative product.

## 1. Introduction

In recent years, the connection between food and medicine has become increasingly closer. Scientific research has shown that certain foods contain significant nutrients and medicinal value, which not only provide the essential nutrients required by the body but also help in the treatment of various diseases to some extent [1]. As a result, “food therapy” is gradually being regarded by more people as a feasible supplement or alternative to traditional drug treatments [2]. In 1996, the European Union launched the European Science of Functional Foods (FUFOSE) research program, clarifying the concept of functional foods. It is believed that consuming functional foods not only meets consumers’ demands for health products but also effectively reduces the occurrence of various diseases [3]. According to the data of 2018, its market was approximately 170 billion US dollars, and this value approximately doubled within five years. It is expected to reach 300 billion US dollars by 2025. This market is mainly driven by North America, Europe, Asia, and the Pacific region [4]. In recent years, many researchers have extracted bioactive substances from edible herbal plants and applied them to the development of functional foods, thus adding more value to these products [5].

Buddha’s hand, named for its shape resembling fingers, has mature fruits with a bright yellow skin and a refreshing, crisp taste accompanied by a strong fragrance. In some regions, Buddha’s hand is processed into dried fruit or juice for consumption [6]. Additionally, Buddha’s hand is rich in various bioactive compounds, including flavonoids, coumarins, essential oils, polysaccharides, amino acids, and trace elements, demonstrating a wide range of pharmacological activities such as antibacterial, anti-inflammatory, antioxidant, anticancer, cough-suppressing, asthma-relieving, blood lipid-lowering, and blood sugar-lowering effects [7].

Galangal is widely used in the fields of medicine, food, and spices, with some regions considering it an essential ingredient in cooking [8]. Research shows that galangal is rich in bioactive components such as essential oils and monoterpene flavonoids, demonstrating significant functions, including antioxidant, analgesic, anti-inflammatory, and digestive-promoting effects [9].

Citron is a plant with both food and medicinal value, and it has a long history of use in various traditional medical systems around the world [10]. In the West, it is often processed into candied fruit to relieve symptoms of indigestion. In the Mediterranean region, citron is widely used for its insect-repellent, antiviral, and soothing properties. In Persia, citron syrup is used as a treatment for migraines. Along the coastal regions of Cameroon, citron is employed to treat respiratory diseases. As modern pharmacological research continues to advance, more of citron’s potential benefits are being revealed, highlighting its significant development value [11].

It is worth noting that Buddha’s hand, citron, and galangal, due to their significant antioxidant, immune-boosting, anti-inflammatory, and sleep-improving properties, align closely with the core concept of functional food development. However, a thorough review of the relevant literature reveals that there is currently a lack of in-depth research on optimizing the brewing process parameters for Buddha’s hand, citron, and galangal wine (BCG wine).

Yeast can break down components such as sugars, fats, proteins, and fibers in fruits through biotransformation, producing bioactive compounds beneficial to the human body. However, in the winemaking process, precise control of fermentation parameters is crucial. Key parameters include yeast inoculation amount, fermentation time, pH, extract content, fermentation temperature, and SO_2_ addition. In our preliminary study, by screening seven potential fermentation parameters, we identified four parameters (pH, fermentation time, extract content, and yeast inoculation amount) that have a significant impact on the quality of BCG wine [12,13,14]. To further optimize these parameters, this study applied the Box-Behnken design (BBD) combined with Response Surface Methodology (RSM) to construct a polynomial model that predicts and analyzes the effects of the selected independent variables on wine quality [15].

Therefore, this study aims to investigate the impact of fermentation conditions on BCG wine and optimize them, laying the foundation for the large-scale and commercial production of this beverage, ultimately benefiting human health and longevity.

## 2. Materials and Methods

### 2.1. Yeast Strains and Materials

Two commercial yeast strains (active dry yeasts), namely SY and RW, were obtained from Angel Yeast (Yichang, Hubei, China). They are typically used to make fruit wine and produce a good aroma. Both of these yeast strains are suitable for fruit wine fermentation.

The commercially mature Buddha’s hand fruits and citron fruits are produced in the Buddha’s hand plantations in Leshan City, Sichuan Province, China. The galangal is provided by Sichuan Chinese Herbal Decoction Pieces Factory Co., Ltd. in Chengdu, China.

The white granulated sugar was supplied by Lotus Group Co., Ltd. (Zhoukou, China). Food-grade sodium bicarbonate is provided by Jincheng Chemical Co., Ltd. (Huainan, China). The rutin standard (batch number: T10J10Z90356), gallic acid standard (batch number: DSTDB003601), and anhydrous glucose standard (batch number: RP10310) were provided by Yuanye Biotechnology Co., Ltd. (Shanghai, China). 3,5-Dinitrosalicylic acid, Folin-Ciocalteu reagent, sodium hydroxide, sodium carbonate, aluminum nitrate, and sodium nitrite were supplied by Jindong Tianzheng Fine Chemical Reagent Factory (Tianjin, China). All these reagents were of analytical grade.

### 2.2. The Preparation of Fermented Wine

After cutting fresh Buddha’s hand, citron, and galangal into small pieces, they were crushed using a juicer (YK-JP01, Zhongshan Jinwei Electric Co., Ltd., Zhongshan, China) until the pulp reached a coarse, granular consistency. Then, 2.4 g/L of pectinase with an activity of 30,000 U/g (Nanning Pangbo Bioengineering Co., Ltd., Nanning, China) was added to the fruit pulp and enzymatically hydrolyzed at 50 °C for 3 h. The clarified solution was then transferred into a 1000 mL fermentation vessel, which was pasteurized at 60 °C for 15 min.

During the fermentation process, single-strain fermentation is adopted. The pre-activated yeast (according to the operation manual provided by Angel Yeast Company, add 10 times the 2% sugar water by mass ratio, activate at 38 °C for 20 min. In addition, it can be used for fermentation after a large number of bubbles are produced in the bacterial liquid) and the pre-treated fruit pulp were added to the fermentation container with a one-way exhaust valve. This equipment is provided by Yantai Diboshi Brewing Equipment Co., Ltd. in Yantai, China. Adjust the extract content using 4.4% (*w*/*w*) white granulated sugar and adjust the pH with sodium bicarbonate. Fermentation was carried out at a constant temperature of 28 °C for 12 days under conditions of constant temperature, sealing, and stirring, followed by 4 °C for 30 days. After completion, parameters such as alcohol content, reducing sugars, total flavonoids, total phenols, and total acids were measured.

### 2.3. Single-Factor Experimental Design of Fermentation Conditions

The single-factor experimental design was employed to analyze the quality of the fermented wine from Buddha’s hand, citron, and galangal. The variable parameters included yeast inoculation amounts (0.2, 1, 2, 4, 6, 8, and 10 g/L), fermentation temperatures (16, 20, 24, 28, 32, 36, and 40 °C), extract contents (17, 19, 21, 23, 25, 27, and 29 °Bx), and pH levels (2.6, 3.0, 3.4, 3.8, 4.2, 4.6, and 5.0). The fermentation conditions of the BCG wine were determined based on indicators such as alcohol content, reducing sugars, total phenols, total acids, total flavonoids, and total phenolic content.

### 2.4. Box-Behnken Design and Analysis

The BBD was employed, with three factors and three levels set for each variable for fermentation optimization. Based on the results of the single-factor experiments, the optimal fermentation conditions for the BCG wine were determined through response surface experiments. The independent variables having the greatest influence on the experimental design were the extract content (25, 27, 29 °Bx), the yeast inoculation amount (6, 8, 10 g/L), the pH (3.4, 3.8, 4.2), and the fermentation temperature (24, 28, 32 °C). BBD was utilized to evaluate the combined effects of the four independent variables. A total of 29 experiments were conducted, among which the central point (with five repetitions) was used for the optimization of the fermentation conditions. The range of the independent variables in the design was specified as three levels, encoded as −1, 0, and +1, and the measured dependent variable was the comprehensive score (Y). The levels of the independent variables and the design matrix are presented in Table 1. All the experimental data obtained from the designed experiments were fitted with a quadratic model using Equation (1):

(1) $$\Upsilon =\beta_{0}+\sum_{i=1}^{4}\beta_{i}{Χ}_{i}+\sum_{i=1}^{4}\beta_{\mathrm{ii}}{Χ}_{i}^{2}+\sum_{j=i+1}^{4}\sum_{i=1}^{4}\beta_{\mathrm{ij}}{Χ}_{i}{Χ}_{j}$$

In this context, Y denotes the predicted response value, while A, B, C, and D, respectively, correspond to the four independent variables of initial sugar content, yeast inoculation amount, pH, and fermentation temperature. β_0_ indicates the intercept; β_i_ represents the linear coefficient; β_ii_ stands for the interaction coefficient; β_ij_ is the quadratic coefficient; and Y is the response value.

### 2.5. Assessment of the Quality Parameters of Fermented Wines

#### 2.5.1. Physicochemical Analysis

The contents of alcohol, pH, reducing sugar content, and total acid were determined according to the standard GB/T 15038-2006 [16], “Analytical methods for wine and fruit wine”.

#### 2.5.2. Determination of Total Flavonoid Content

Under the findings of Sun et al. [17], the total flavonoid content was quantified using the aluminum chloride colorimetric method. Absorbance measurements were conducted at 510 nm utilizing an ultraviolet-visible spectrophotometer (UV2600, Shanghai Sunny Hengping Scientific Instrument Co., Ltd., Shanghai, China). The standard curve demonstrated a robust linear correlation at 510 nm (*Y* = 0.2724*X* + 0.0044, *R*^2^ = 0.9984), where *Y* denotes absorbance and *X* represents rutin concentration (mg/L).

#### 2.5.3. Determination of Reducing Sugar Content

According to Sun et al. [17], the concentration of reducing sugars was quantified using the 3,5-dinitrosalicylic acid method. Absorbance measurements were conducted at 540 nm with a UV-visible spectrophotometer (UV2600, Shanghai Shunyu Hengping Scientific Instrument Co., Ltd., Shanghai, China). The standard curve demonstrated a linear correlation at this wavelength (*Y* = 432.5*X* + 0.0243, *R*^2^ = 0.9998), where *Y* denotes absorbance and *X* indicates glucose concentration (mg/L).

#### 2.5.4. Determination of Total Phenolic Content

In accordance with Sun et al. [17], the total phenolic content was quantified using the Folin-Ciocalteu method. Absorbance measurements were conducted at 760 nm using a UV-visible spectrophotometer (UV2600, Shanghai Shunyu Hengping Scientific Instrument Co., Ltd., China). The standard curve exhibited a linear correlation at this wavelength (*Y* = 9.0789*X* + 0.0669, *R*^2^ = 0.9954), where *Y* denotes absorbance and *X* represents gallic acid concentration (mg/L).

#### 2.5.5. Sensory Evaluation Scoring

In accordance with the sensory evaluation methodology for plum wine established by Sui [18] and the “Analysis Methods for Wine and Fruit Wine” (GB/T 15038-2006) [16], the Food and Biological Engineering Committee of Xihua University authorized this research on sensory assessment. All participants provided informed consent prior to their involvement in the study. A total of ten qualified evaluators (five males and five females, aged between 23 and 43) employed a 100-point scoring system for their evaluations. Each evaluator was in good health and had completed a minimum of 90 h of standardized training before conducting assessments. The evaluators’ results demonstrated that the mean square deviation for color, taste, aroma, and style accuracy was less than 0.05. Prior to the sensory evaluation, equal volumes of BCG wine were dispensed into identical test cups. Each evaluator was assigned to separate testing rooms to mitigate potential interference among them. Furthermore, evaluators rinsed their mouths before commencing formal evaluations. The evaluation criteria are detailed in Table 2.

### 2.6. Analysis of Fermented Wine Aroma Components

In accordance with the detection methodology for component analysis of pitaya as described by Lin et al. [19], minor modifications were implemented to the heating procedure.

Eight milliliters of the sample were transferred into a 20 mL headspace vial, to which 2 g of sodium chloride and 2 µL of 2-octanol were added. The vial was then equilibrated at 40 °C for 15 min. After equilibration, a solid phase microextraction (SPME) needle was inserted into the headspace vial, and adsorption was carried out for 30 min. The sample was then desorbed at 250 °C for 3 min in the injection port.

Chromatographic conditions: An InertCap Wax capillary column (60 m × 0.25 mm × 0.25 μm) was used, and the temperature program was as follows: the initial temperature was set at 40 °C and held for 3 min, then increased at a rate of 5 °C/min to 120 °C, followed by an increase at 8 °C/min to 230 °C, where it was maintained for 10 min. The injection port temperature was set to 250 °C, and the carrier gas flow rate was 1.2 mL/min, using high-purity helium (99.99% purity) as the carrier gas. The injection mode was splitless.

Mass spectrometry conditions: The ion source was electron ionization (EI) with an electron impact energy of 70 eV. The scanning mode was set to scan with an ion source temperature of 230 °C. The mass scan range (*m*/*z*) was from 33 to 450 atomic mass units (AMU).

Qualitative and Quantitative Analysis: Qualitative analysis was performed based on the mass spectra obtained from GC-MS analysis, with reference to the NIST14s.lib spectral library for identification. Retention indices (RI) were also used in conjunction with the spectra for further confirmation. Quantitative analysis was conducted by measuring the relative concentrations of each aroma compound, using 2-octanol as the internal standard.

### 2.7. Data Analysis

#### 2.7.1. Experimental Mathematical Statistics Analysis

All the experiments were run in triplicate, and the data were expressed as the mean ± standard deviation. The figures were plotted using Origin 2020 software (Chicago, IL, USA). Design-Expert version 8.0.6.1 (Stat-Ease Corp., Minneapolis, MN, USA) was used for response surface test design and data analysis.

#### 2.7.2. Entropy Weight Calculation Method

A single indicator cannot fully evaluate the quality of wine. Therefore, sensory scores, total phenol content, total flavonoid content, alcohol content, and reducing sugar content were considered, and the entropy weight method was used to screen the wine with the highest score.

(1) Construct evaluation matrix

(2) $$R_{Χ}=\left[\begin{array}{ccc} {Χ}_{11} & \ldots & {Χ}_{m1}\\ \vdots & \ddots & \vdots \\ {Χ}_{1n} & \ldots & {Χ}_{\mathrm{mn}} \end{array}\right]$$

(2) Standardization of indicators

To eliminate the problem of non-uniformity in the indicators’ scales and units of measurement, it is necessary to standardize each indicator. Standardization of positive indicators:

(3) $$\Upsilon_{\mathrm{ij}}=\frac{{Χ}_{\mathrm{ij}}-\min({Χ}_{\mathrm{ij},\ldots ,}{Χ}_{\mathrm{mj}})}{\max({Χ}_{1j,\ldots ,}{Χ}_{\mathrm{mj}})-\min({Χ}_{1j,\ldots ,}{Χ}_{\mathrm{mj}})}$$

Normalization of negative indicators:

(4) $$\Upsilon_{\mathrm{ij}}=\frac{\max({Χ}_{1j,\ldots ,}{Χ}_{\mathrm{mj}})-{Χ}_{\mathrm{ij}}}{\max({Χ}_{1j,\ldots ,}{Χ}_{\mathrm{mj}})-\min({Χ}_{1j,\ldots ,}{Χ}_{\mathrm{mj}})}$$

The normalization process yields the new matrix:

(5) $$R_{\Upsilon }=\left[\begin{array}{ccc} y_{11} & \ldots & y_{m1}\\ \vdots & \ddots & \vdots \\ y_{1n} & \ldots & y_{\mathrm{mn}} \end{array}\right]$$

(3) The weight of the jth object to be evaluated under the ith evaluation indicator

(6) $${Ρ}_{\mathrm{ij}}=\frac{\Upsilon_{\mathrm{ij}}}{\sum_{j=1}^{n}\Upsilon_{\mathrm{ij}}}(i=1,2,\cdots ,m;j=1,2,\cdots ,n)$$

(4) Entropy value of the ith evaluation indicator

(7) $$e_{i}=-\frac{1}{\ln\;n}\sum_{j=1}^{n}{Ρ}_{\mathrm{ij}}\ln{Ρ}_{\mathrm{ij}}(i=1,2,\cdots ,m;j=1,2,\cdots ,n)$$

(5) Determine the weight

(8) $$W_{i}=\frac{1-e_{\mathrm{ij}}}{\sum_{j=1}^{m}\left(1-e_{\mathrm{ij}}\right)}(i=1,2,\cdots ,m;j=1,2,\cdots ,n)$$

Based on the assigned values of each index calculated by the above formula, the comprehensive score of each group of wines and the influence of different indicators on BCG wines were obtained.

## 3. Results

### 3.1. Results of One-Factor Experiment

**Figure 1:**
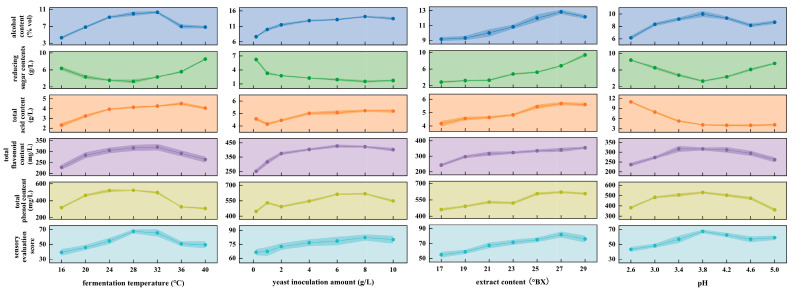
The influence of various factors on wine.

There is a wide variety of fruit wines, each characterized by its unique flavor profile. The color, aroma, and taste presented by these wines largely depend on the quality of the raw materials, the yeasts used, and the specific fermentation processes. Different types of yeast may induce distinct chemical transformations, resulting in variations in phenolic and aromatic compounds [20]. These differences ultimately contribute to the nutritional value and aromatic potential of the final product. The effects of different yeasts and fermentation parameters on the nutritional value and taste of BCG wine are shown in Table 3 and Figure 1.

#### 3.1.1. The Effects of Different Yeasts

Phenolic substances contribute significantly to the quality, conferring a more abundant structural perception and alcohol thickness to the wine while making the palate more refreshing. Meanwhile, interacting with an appropriate quantity of acidic substances and flavonoids, they can activate the distinctive aroma and rich layering of the fruit wine [20,21]. The wine fermented by SY has a higher total phenol content and lower total flavonoid and total acid levels, making the wine body taste more mellow, with a harmonious balance of fruit and wine aroma, thus achieving a higher sensory score. In contrast, the wine fermented by RW has a lower total phenol content while having higher total acid and total flavonoid levels, resulting in an overall taste that is more sour and astringent, yielding a relatively lower sensory score. Additionally, the above results indicate that SY has stronger fermentation vitality and can produce a more suitable alcohol content, making it more suitable as a fermentation yeast for BCG wine.

#### 3.1.2. Effect of Fermentation Temperature on Fermentation

Raising the fermentation temperature can accelerate the fermentation process. However, it also accelerates yeast aging, which leads to a decrease in alcohol content and a loss of volatile compounds, ultimately reducing the overall quality of the wine. Conversely, lower fermentation temperatures extend the fermentation time [22]. Studies have shown that wines produced at 28 °C exhibit the highest contents of total phenols, total flavonoids, and alcohol, along with superior sensory scores.

#### 3.1.3. Effect of the Yeast Inoculation Amount on Fermentation Effect

As the yeast inoculation amount increased, the alcohol content gradually rose. When the yeast inoculation amount reached 8 g/L, the alcohol content, total phenolic content, and sensory score peaked at (14.17 ± 0.29) %vol, (612.52 ± 7.17) mg/L, and (82.3 ± 3.37), respectively. Meanwhile, total acidity and reducing sugar levels tended to stabilize. However, when the inoculation amount exceeded 8 g/L, the excessive yeast rapidly consumed reducing sugars and produced large amounts of metabolic byproducts, which adversely affected the fermentation process [22].

#### 3.1.4. Effect of Extract Content on Fermentation Effect

Sugar serves as a key substrate for fermentation, as yeast converts sugars into alcohol; thus, sugar concentration directly influences alcohol content. However, higher sugar concentrations increase the osmotic pressure of the fermentation medium, which can inhibit yeast growth and reproduction, prolong the fermentation period, and lead to the formation of byproducts such as higher alcohols and succinic acid, contributing to a more bitter taste [14]. At an extract content of 27 °Bx, the alcohol content reached its maximum, accompanied by a high total phenolic content, lower contents of total flavonoids and total acidity, and an overall improved flavor profile, resulting in the highest sensory evaluation score.

#### 3.1.5. Effect of pH on Fermentation Effect

Extremely high or low pH levels are unfavorable for fermentation. As pH increases, the alcohol content initially rises and then declines. At pH 3.8, the reducing sugar content was the lowest, while the alcohol content, total phenolic content, total flavonoid content, and sensory evaluation score reached their highest values: (10.0 ± 0.5) %vol, (529.54 ± 10.24) mg/L, (316.69 ± 7.52) mg/L, and (67.4 ± 2.41), respectively. Furthermore, Tongwei Guan et al. found that SY jujube wine had a better taste at a pH of 3.15 [23], while Chenglin Zhu et al. used a pH of 4.0 when studying the metabolomics of Guang’an Honey Pear Apple wine [24]. Therefore, it indicates that SY has good adaptability between 3.15 and 4.0, and our research results are consistent with the above findings, which precisely supports this point.

### 3.2. Response Surface Test Results and Analysis

#### 3.2.1. The Outcomes of the Entropy Weight Calculation and Response Surface Methodology Experimental Designs

Response surface experiments were conducted based on Table 1, and the results are shown in Table 4.

Based on the method described in Formula (1), the matrix was constructed, yielding the following result:

$$R_{x}=\left[\begin{array}{ccccc} 334.56 & 669.16 & 13 & 5.47 & 89.5\\ 336.76 & 672.27 & 15 & 4.83 & 85.2\\ \vdots & \vdots & \vdots & \vdots & \vdots \\ 342.87 & 683.11 & 18 & 4.06 & 81.5 \end{array}\right]$$

The positive indicators include alcohol content, sensory score, total phenol content, and total flavonoid content, while the negative indicator is reducing sugar. Upon standardizing matrix R_x_, the following result was obtained:

$$R_{Y}=\left[\begin{array}{ccccc} 0.05\ldots 65 & 0.06\ldots 32 & 0.00\ldots 01 & 0.013\ldots 31 & 0.86\ldots 82\\ 0.28\ldots 01 & 0.25\ldots 77 & 0.40\ldots 01 & 0.45\ldots 96 & 0.42\ldots 13\\ \vdots & \vdots & \vdots & \vdots & \vdots \\ 0.93\ldots 46 & 0.90\ldots 78 & 1.00\ldots 01 & 0.97\ldots 56 & 0.04\ldots 14 \end{array}\right]$$

Calculate the entropy value P_j_ of each indicator according to Formula (3):

$$P_{j}=\left(\begin{array}{ccccc} 0.88\ldots 82 & 0.91\ldots 04 & 0.90\ldots 65 & 0.91\ldots 78 & 0.89\ldots 09 \end{array}\right)$$

The entropy weight W_i_ of each index was calculated based on Formula (4):

$$W_{i}=\left(\begin{array}{ccccc} 23 & 18 & 19 & 17 & 23 \end{array}\right)$$

The calculated weight ratios are as follows: total flavonoid content (0.23), total phenol content (0.18), alcohol content (0.19), reducing sugar content (0.17), and sensory score (0.23). Subsequently, by substituting the weights Wi into the score set, the comprehensive score was derived as follows:

$$Q_{\mathrm{ij}}=R_{x}\times W_{i}=\left[\begin{array}{ccccc} 334.56 & 669.16 & 13 & 5.47 & 89.5\\ 336.76 & 672.27 & 15 & 4.83 & 85.2\\ \vdots & \vdots & \vdots & \vdots & \vdots \\ 342.87 & 683.11 & 18 & 4.06 & 81.5 \end{array}\right]\times (\begin{array}{c} 23\\ 18\\ 19\\ 17\\ 23 \end{array})$$

Thus, the entropy-weighted comprehensive scores for the 29 experimental groups were obtained. The detailed response surface design scheme and corresponding comprehensive scores are presented in Table 5.

#### 3.2.2. Establishment of Fitting Model and Analysis of Variance

The quadratic response surface regression analysis was applied to the data presented in Table 5 to derive the quadratic multiple regression equation for the total score, as shown in Equation (9).

y = −6901.54615 + 245.53417A + 128.48958B – 1174.68021C + 61.89844D – 1.58250AB + 0.440625AC + 0.061875AD – 3.96875BC + 0.265625BD – 2.22344CD – 4.33552A^2^ – 4.51396B^2^ – 145.02083C^2^ – 1.00357D^2^ (9)

Table 6 delineates the results of variance and significance analysis for the regression models pertaining to each factor and the comprehensive score. The adequacy and predictability of these models are assessed based on the coefficient of determination (R^2^) and the adjusted coefficient of determination (adj. R^2^) [25]. Concerning the comprehensive score, R^2^ and adj. R^2^ values are 0.9577 and 0.9153, respectively, indicating that the model exhibits an excellent fit by accounting for over 90% of the variance in the response variable under investigation. Given that R^2^ and adj. R^2^ yield values of 0.7693 and 0.9153, respectively, in PV results, it is evident that this model demonstrates superior fitting capabilities by explaining more than 77% of the variance in the studied response variable [26].

To support the sufficiency and model fit data, lack-of-fit tests were conducted. In this study, the *p*-value associated with “lack-of-fit” was found to be 0.0674. These findings suggest that “lack-of-fit” does not reach statistical significance (*p* > 0.05), thereby affirming model reliability [27]. Consequently, our model effectively elucidates relationships between comprehensive scores and factors such as extract content, yeast inoculation amount, and pH value, as well as fermentation temperature, while providing improved predictions for composite scores under varying fermentation conditions.

In this model, the primary terms A, C, and D had significant effects; B had highly significant effects; the interaction term AB had significant effects; the secondary terms A^2^, B^2^, C^2^, and D^2^ had highly significant effects; and the other terms were not significant, indicating that the relationship between the selected factors and the response values was not a simple linear relationship. Furthermore, the ranking of the influence of each factor on fermented wine is presented as follows: yeast inoculation amount (B) > extract content (A) > fermentation temperature (D) > pH (C).

**Figure 2:**
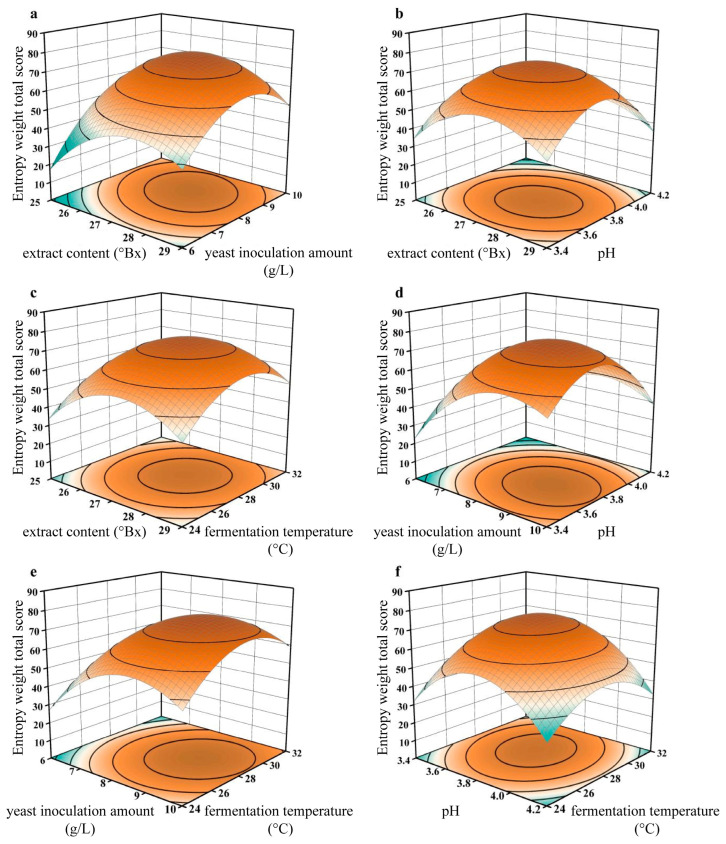
Two-way interaction of extract content: (**a**) extract content and yeast inoculation amount, (**b**) extract content and pH, (**c**) extract content and fermentation temperature, (**d**) yeast inoculation amount and pH, (**e**) yeast inoculation amount and fermentation temperature, (**f**) pH and fermentation temperature.

The influence results of the interaction among various factors on the wine are shown in Figure 2. The steeper the gradient, the greater the influence. The steep gradient in the interaction map of extract content and yeast inoculation amount indicates that the interaction is significant.

#### 3.2.3. Response Surface Validation

After being analyzed by Design Expert 8.0 software and predicted by the model, the optimal fermentation conditions for BCG wine were an extract content of 27.13 °Bx, 8.67 g/L of yeast inoculation amount, 3.75 pH, and 28.67 °C of fermentation temperature. Under these optimized conditions, the predicted composite score was 77.48. To verify whether the model was accurate and reliable, three replications were carried out with the modified process conditions, and the results are shown in Table 7. The results indicated that the mean composite score of the BCG wine was 77.87. Its RSD was 0.85%, which was in good agreement with the predicted value, indicating that the process parameters obtained based on the Box-Behnken experimental This indicates that the process parameters obtained based on the Box-Behnken test are reliable and have practical application value. The results of a content determination under the extraction process conditions also showed that the sensory scores of the optimized fermented wine were significantly improved compared to the original process conditions with no change in nutrients, which further indicated that the process parameters had application value.

### 3.3. Analysis of Aroma Compounds in BCG Wine

**Figure 3:**
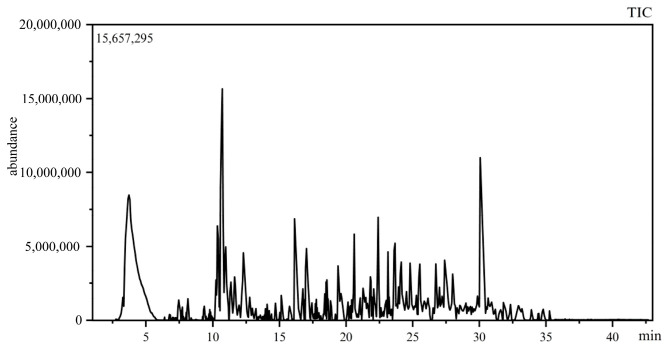
GC-MS total ion flow diagrams of BCG wine.

Based on Table 8 and Figure 3, a total of 67 aromatic compounds were identified. These include 22 esters, with a relative content of 19.787%; 1 acid, with a relative content of 1.59%; 20 alcohols, with a relative content of 47.786%; 3 ethers, with a relative content of 0.906%; 3 ketones, with a relative content of 1.495%; 2 aldehydes, with a relative content of 1.25%; and 18 olefins, with a relative content of 14.713%. Overall, alcohols are the most abundant in terms of both variety and content, followed by esters and olefins.

According to the results of the aroma threshold value calculations, 15 components have an OAV greater than 1, which directly impacts the overall flavor of BCG wine. The most significant contributor is linalool, followed by alpha-terpinene and alpha-bergamotene. As a result, the predominant aroma of the wine is fruity, with hints of lemon and pinewood. Additionally, 18 components have an OAV between 1 and 0.1, which imparts floral, fruity, and alcoholic aromas, thereby enhancing the overall complexity and depth of the wine.

## 4. Discussion

Many fruits with considerable nutritional and therapeutic potential—such as Buddha’s hand, citron, and galangal—remain significantly underutilized. However, the current research predominantly emphasizes direct consumption or juicing, with only a few studies exploring essential oils. Furthermore, a limited number of studies have reported on the application of essential oils. Nevertheless, numerous food processing techniques remain underexplored, with alcoholic beverages emerging as a particularly promising research area.

Currently, numerous studies have demonstrated the feasibility of utilizing fruits with therapeutic properties in the production of alcoholic beverages. For instance, Attri et al. integrated ginger juice into the production of pear wine, resulting in a ginger-pear beverage with significantly enhanced flavor, extended shelf life, and increased value due to the well-known therapeutic properties of ginger [28]. Similarly, Johnson applied enological fermentation techniques to optimize mulberry wine production, achieving elevated selenium concentrations [29]. However, to date, there have been no published reports on the fermentation of beverages using Buddha’s hand, citron, and galangal.

This study employed SY as the fermentation agent and a mixture of Buddha’s hand, citron, and galangal as the raw materials. An integrated optimization approach combining entropy weight analysis and response surface methodology was employed to refine fermentation parameters. The optimal conditions were identified as follows: yeast inoculation amount of 8.6 g/L, pH of 3.75, fermentation temperature of 28.67 °C, and extract content of 27.13 °Bx. Under these conditions, the brewed wine exhibited excellent taste characteristics and contained abundant nutritional components. This study addressed a significant gap in the field and provided novel insights into the utilization of edible fruits with therapeutic properties. Furthermore, it established a solid foundation for future research and industrial production of BCG wine.

RSM is a systematic approach that integrates mathematical and statistical techniques. It is widely employed to model and analyze problems involving multiple variables that influence the associated responses. In addition, as an optimization technique, it takes into account random errors, thereby overcoming the limitations of traditional optimization methods, and has been widely applied in various fields. Currently, many researchers have employed RSM to optimize brewing processes. Gong et al. optimized the production process of noni wine using RSM [30]. Yan et al. optimized the fermentation temperature, pH, and inoculum size of plum wine, using taste as the response variable [31]. Liu Y et al. used alcohol content as the response variable to optimize the fermentation temperature, SO_2_ addition rate, and initial sugar content of carrot-pomegranate fermented wine [32]. Wu et al. conducted an RSM-based optimization of kiwi wine fermentation conditions (temperature, pH, and extract content), using alcohol content as the response variable [33]. According to current studies, brewing conditions are mostly optimized based on a single indicator, such as alcohol content or taste. However, when fruits with therapeutic properties are used for alcoholic fermentation, it is essential to consider not only alcohol content and sensory quality but also the nutritional components of the product.

The EWM is a comprehensive approach that accounts for multiple indicators. This method integrates and calculates various metrics to derive a composite score. It can be combined with RSM by using the composite score as the response variable to optimize processing conditions. As such, EWM enables the evaluation of a broader range of criteria, and its feasibility has already been demonstrated in existing studies.

Gai et al. incorporated seven different indicators into their research framework and applied EWM to comprehensively evaluate these metrics in order to optimize the extraction process of Elaeagnus pungens [34]. Huang et al. used EWM to calculate information entropy and determine the weights of five specific indicators, thereby deriving a comprehensive index to evaluate the efficiency of high-speed counter-current chromatography; this composite index was subsequently used as the response variable for process optimization [35]. Sun et al. successfully optimized the extraction method of total flavonoids from *Abelmoschus manihot* flowers by applying a combined approach of EWM and RSM [36]. To date, no published studies have reported the use of EWM in combination with RSM to optimize the fermentation process of fruit wines.

In this study, we applied the EWM to the preparation of fruit wine for the first time. Five indicator—alcohol content, sensory attributes, reducing sugar, total flavonoids, and total polyphenols—were assigned weights using EWM. The corresponding weights were as follows: alcohol content (0.19), sensory attributes (0.23), reducing sugar (0.17), total flavonoids (0.23), and total polyphenols (0.18). These results indicate that total flavonoid content and sensory quality have relatively greater influences on the overall evaluation.

The coefficient of determination R^2^ was 0.9577, and the Adj. R^2^ was 0.9153, suggesting that the model is highly reliable and that the regression can effectively predict the response values. Therefore, it is feasible to optimize the fruit wine fermentation process by combining the entropy weight method with response surface methodology. This study provides a novel optimization framework for the fermentation process of fruit wines and the production of functional fermented beverages.

In this study, we used HS-SPME-GC-MS to analyze the flavor compounds of BCG wine. The results indicated that 13 components possess an OAV greater than one, which directly impacts the overall flavor of BCG wine. The most significant contributor is linalool, followed by ethyl hexanoate and cineole. As a result, the predominant aroma of the wine is fruity, with hints of lemon and herbs. Additionally, nine components have an OAV between 1 and 0.1, which impart floral, brandy, and alcoholic aromas, thereby enhancing the overall complexity and depth of the wine. Furthermore, findings indicate that the components present at relatively high concentrations are known to possess potential anti-inflammatory, antioxidant, and sleep-promoting properties [37,38,39]. This not only enhances the added value of BCG wine but also provides a solid foundation for further investigation into its underlying mechanisms.

## 5. Conclusions

This study represents the first systematic investigation into the brewing process of Buddha’s hand, citron, and galangal wine. The results indicate that using SY as the yeast, with a yeast inoculation amount of 8.6 g/L, pH of 3.75, fermentation temperature of 28.67 °C, and extract content of 27.13 °Bx, produces a wine with an excellent taste, distinctive flavor profile, and abundant nutritional components. Furthermore, this study marks the first application of the entropy weight method in conjunction with RSM for optimizing the brewing process of fermented wines. By incorporating five key indicators simultaneously into the evaluation framework, both sensory quality and nutritional content are maximized. Upon validation, the model demonstrated a high determination coefficient and low relative standard deviation, confirming its reliability and practical applicability.

Based on the aforementioned findings, we performed a comprehensive analysis of the flavor compounds in Buddha’s hand, citron, and galangal wine. The results revealed that the predominant aroma of the wine is fruity, with subtle notes of lemon and herbs. Additionally, the wine exhibits certain floral, brandy-like, and alcoholic aromas, which collectively contribute to its overall complexity and depth. Notably, our study demonstrates that the components present at relatively high concentrations possess potential anti-inflammatory, antioxidant, and sleep-promoting properties. These insights not only enhance our understanding of the wine’s chemical profile but also provide valuable guidance for optimizing the production process of Buddha’s hand, citron, and galangal wine.

## Figures and Tables

**Table 1:** The coded and actual values of factors in the BBD.

**Factor**	**Name**	**Low Actual**	**High Actual**	**Low Coded**	**High Coded**
A	extract content	25	29	−1	1
B	yeast inoculation amount	6	10	−1	1
C	pH	3.4	4.2	−1	1
D	fermentation temperature	24	32	−1	1
**Response**	**Name**	**Observed**	**Min**	**Max**	**Mean**
Y	Total score	29	22.27	74.30	48.29

**Table 2:** Sensory rating scale.

Sensory Indicator	Evaluation	Score
Color (20 Points)	1. Clear, transparent, shiny, and pleasing to the eye coordination	20
	2. Clear and transparent	18–19
	3. Micromixing and loss of light	15–17
	4. Opacity and light loss	0–14
Smell (30 Points)	1. Fruit fragrance and wine aroma are rich in coordination, with a pleasant aroma	28–30
	2. Fruit and wine fragrance, with aroma	24–27
	3. Fruit and wine aroma is light, and aroma is insufficient	20–23
	4. Fruit and wine fragrances are insufficient or have strange fragrances with a pungent smell	0–21
Taste (40 points)	1. Wine is full and mellow, comfortable and pleasant, and pure without miscellaneous	38–40
	2. The wine body is soft and refreshing, and the sweet and sour taste is appropriate	30–37
	3. Wine is not rich, slightly bitter, and slightly sour	25–29
	4. Wine is bitter, astringent, sour, and smelly	0–25
Style (10 points)	1. Outstanding style, have a unique style	10
	2. Typical, clear, and good in style	9
	3. General style	7–8
	4. Loss of fruit wine typicality	0–6

**Table 3:** The influence of different yeasts on wine production.

Yeast Type	Alcohol (%vol)	Reducing Sugar (g/L)	Total Acid (g/L)	Total Flavonoids (mg/L)	Total Phenols (mg/L)	Sensory Evaluation
SY	10 ± 0.13	3.30 ± 0.38	5.0183 ± 0.31	317.91 ± 3.12	528.81 ± 7.95	66.7 ± 4.11
RW	9.4 ± 0.27	3.10 ± 0.41	5.5812 ± 0.27	325.45 ± 2.98	493.16 ± 6.11	59.1 ± 3.09

**Table 4:** Measurement results of response surface test indicators.

Std	Flavonoid Content (mg/L)	Total Phenol Content (mg/L)	Alcohol Content (%vol)	Reducing Sugar Content (g/L)	Sensory Evaluation
1	334.56 ± 11.09	669.16 ± 9.1	13 ± 0.5	5.47 ± 0.35	89.5 ± 4.8
2	336.76 ± 11.03	672.27 ± 8.2	15 ± 0.87	4.83 ± 0.26	85.2 ± 5.3
3	341.34 ± 7.17	679.23 ± 11.49	17 ± 0.5	4.69 ± 0.27	81.1 ± 6.1
4	338.01 ± 7.06	675.15 ± 7.12	16 ± 0.87	4.71 ± 0.13	83.3 ± 6.4
5	336.07 ± 9.71	672.97 ± 8.1	15 ± 0.87	4.85 ± 0.28	85.3 ± 4.9
6	334.21 ± 10.72	670.32 ± 10.91	14 ± 0.87	5.25 ± 0.16	88.8 ± 5.6
7	338.95 ± 9.84	678.86 ± 10.08	16.5 ± 0.5	4.62 ± 0.17	82.3 ± 7.0
8	334.27 ± 7.22	670.75 ± 8.71	14 ± 0.87	5.27 ± 0.33	88.9 ± 6.3
9	336.34 ± 9.17	672.31 ± 7.15	15 ± 0.87	4.87 ± 0.22	85.1 ± 5.9
10	338.73 ± 7.42	675.48 ± 11.11	16 ± 0.87	4.70 ± 0.36	83.7 ± 4.6
11	338.15 ± 9.40	672.12 ± 10.55	15.5 ± 0.5	4.77 ± 0.15	85.3 ± 6.6
12	340.75 ± 8.60	679.56 ± 9.11	17 ± 0.87	4.63 ± 0.27	81.9 ± 5.7
13	334.82 ± 7.21	669.22 ± 11.63	13 ± 0.5	5.44 ± 0.35	89.2 ± 4.6
14	340.48 ± 10.26	679.98 ± 10.95	17 ± 0.5	4.68 ± 0.27	81.8 ± 5.9
15	334.67 ± 8.98	669.53 ± 9.07	13 ± 0.5	5.45 ± 0.17	90.3 ± 6.0
16	338.2 ± 7.91	675.95 ± 7.32	16 ± 0.87	4.7 ± 0.32	83.4 ± 6.6
17	334.07 ± 8.95	669.31 ± 8.44	13.5 ± 0.5	5.34 ± 0.27	90.8 ± 7.0
18	336.89 ± 10.97	672.44 ± 10.1	15.5 ± 0.5	4.79 ± 0.12	85.2 ± 6.6
19	334.47 ± 8.71	669.59 ± 11.76	13 ± 0.87	5.49 ± 0.29	89.7 ± 6.7
20	336.28 ± 8.66	672.23 ± 8.45	15 ± 0.87	4.8 ± 0.31	85.3 ± 4.8
21	334.51 ± 11.17	668.05 ± 8.55	13 ± 0.5	5.42 ± 0.16	90.1 ± 6.8
22	338.22 ± 9.31	675.37 ± 10.98	16 ± 0.87	4.79 ± 0.15	83.7 ± 5.7
23	334.48 ± 9.01	670.64 ± 11.89	14 ± 0.5	5.31 ± 0.26	88.6 ± 4.9
24	341.48 ± 9.13	679.71 ± 8.2	17 ± 0.87	4.61 ± 0.31	81.2 ± 7.2
25	342.48 ± 7.25	681.58 ± 9.64	18 ± 0.5	4.19 ± 0.21	81.9 ± 5.8
26	343.34 ± 11.28	683.93 ± 9.89	18 ± 0.5	4.03 ± 0.34	82.1 ± 6.8
27	342.82 ± 10.37	683.14 ± 11.9	17.5 ± 0.5	4.14 ± 0.17	82.2 ± 6.1
28	343.48 ± 8.45	684.69 ± 8.04	18 ± 0.5	4.18 ± 0.23	81.3 ± 4.9
29	342.87 ± 9.42	683.11 ± 9.97	17.5 ± 0.5	4.06 ± 0.33	81.5 ± 7.6

**Table 5:** The BBD matrix and responses. Y is the result calculated according to the weight.

Std	Independent Variable				Response
	A	B	C	D	Y
1	−1	−1	0	0	22.32
2	1	−1	0	0	36.12
3	−1	1	0	0	54.6
4	1	1	0	0	43.08
5	0	0	−1	−1	35.17
6	0	0	−1	−1	27.44
7	0	0	−1	1	50.01
8	0	0	−1	1	28.05
9	−1	0	0	−1	34.42
10	1	0	0	−1	46.27
11	−1	0	0	1	42.24
12	1	0	0	1	56.07
13	0	−1	−1	0	22.67
14	0	1	−1	0	55.04
15	0	−1	1	0	25.09
16	0	1	−1	0	44.76
17	−1	0	−1	0	27.73
18	1	0	−1	0	39
19	−1	0	−1	0	22.79
20	1	0	−1	0	35.47
21	0	−1	0	−1	22.98
22	0	1	0	−1	43.83
23	0	−1	0	1	27.28
24	0	1	0	1	56.63
25	0	0	0	0	71.47
26	0	0	0	0	78.46
27	0	0	0	0	73.36
28	0	0	0	0	76
29	0	0	0	0	74.66

**Table 6:** ANOVA and significance test.

Source	Sum of Squares	df	Mean Square	F Value	*p*-Value Prob > F	Significant
Model	8551.82	14	610.84	22.62	<0.0001	Significant
A-extract content	224.55	1	224.55	8.32	0.0120	*
B-yeast inoculation amount	1668.05	1	1668.05	61.77	<0.0001	**
C-pH	176.49	1	176.49	6.54	0.0228	*
D-fermentation temperature	209.75	1	209.75	7.77	0.0145	*
AB	160.28	1	160.28	5.94	0.0288	*
AC	0.497	1	0.4970	0.0184	0.8940	
AD	0.9801	1	0.9801	0.0363	0.8516	
BC	40.32	1	40.32	1.49	0.2419	
BD	18.06	1	18.06	0.6689	0.4271	
CD	50.62	1	50.62	1.87	0.1925	
A^2^	1950.8	1	1950.80	72.24	<0.0001	**
B^2^	2114.68	1	2114.68	78.31	<0.0001	**
C^2^	3492.29	1	3492.29	129.32	<0.0001	**
D^2^	1672.41	1	1672.41	61.93	<0.0001	**
Residual	378.06	14	27.00			
Lack of fit	350.05	10	35.00	5.00	0.0674	Not significant
Pure error	28.02	4	7.00			
Cor total	8929.89	28				
R^2^ = 0.9577				Adj. R^2^ = 0.9153		
C.V.% = 11.84				Adeq Precision = 15.4771		

Note: “**” is the highly significant level (*p* ≤ 0.01), and “*” is the significant level (0.01 < *p* ≤ 0.05).

**Table 7:** Experimental results of the optimal process verification.

	1	2	3
Flavonoid content (mg/L)	342.93	341.98	342.05
Total phenol content (mg/L)	683.26	683.18	683.97
Alcohol content (%vol)	18	18	18
Reducing sugar content (g/L)	4.16	4.21	4.22
Sensory evaluation	83.4	84.2	84.3
Composite score	78.25	77.11	78.25
Average score	77.87		
RSD (%)	0.85%		

**Table 8:** Analysis of aroma components of BCG wine.

Category	Aroma Component	Retention Time (min)	Concentration (mg/mL)	Description of Aroma	OAV
Alkenes	Cyclofenchene	22.31	0.08 ± 0.01	-	-
	Myrcene	14.67	140.75 ± 1.21	Citrus, herbal	4.67
	Ectoine	10.08	1.36 ± 0.13	-	-
	Gamma-terpinene	12.92	0.93 ± 0.08	-	-
	Alpha-pinene	8.79	0.89 ± 0.03		-
	Terpinolene	17.21	34.1 ± 1.91	Citrus, herbal	0.43
	Para-menthatriene	16.82	1.46 ± 0.23	Peppermint	0.15
	Alpha-Terpinene	12.95	890.20 ± 4.33	Citrus, pine wood	8.9
	Camphene	4.59	2.09 ± 0.12	-	-
	Alpha-bergamotene	15.18	148 ± 2.94	Citrus, floral	7.4
	Alpha-caryophyllene	22.83	16.07 ± 1.15	Spicy, woody, pepper	0.54
	Beta-selinene	23.25	4.63 ± 0.36	-	-
	Beta-bisabolene	27.93	0.76 ± 0.07	-	-
	Farnesene	28.35	0.88 ± 0.01	-	-
	Gamma-cadinene	23.92	4.85 ± 0.25	-	-
	Beta-calacorene	26.12	1.68 ± 0.32	-	-
	O-cymene	12.18	0.68 ± 0.11	-	-
	P-cymene	12.45	2.93 ± 0.17	-	-
Aldehydes	Citral	11.18	2.26 ± 0.26	Citrus, Lemon	2.26
	5-Hydroxymethylfurfural	32.32	53.6 ± 0.45	-	-
Alcohols	Ethanol	3.58	793,300 ± 526	Alcohol	0.79
	Isobutanol	7.45	672.23 ± 9.12	-	-
	3-Methyl-1-butanol	10.32	20,230 ± 117	Floral, bitterness	0.67
	Cineole	10.65	87.85 ± 4.33	Peppermint, Herbs	1.76
	2-Ethylhexanol	17.92	1820.12 ± 97.21	Floral, fruit, sweets	3.64
	2,3-Butanediol	19.45	7400 ± 65.32	-	-
	Linalool	19.38	78.51 ± 5.79	Citrus, floral fragrance, and sweet rose scent	9.75
	Fenchol	20.12	28.19 ± 2.33	Floral, pine wood, citrus	1.41
	Terpinen-4-ol	20.58	1.05 ± 0.45	-	-
	4-Isopropenyl-1-methylcyclohexanol	21.12	1.04 ± 0.06	-	-
	Furfuryl alcohol	21.32	221.93 ± 9.79	-	-
	1-Nonanol	21.87	63.35 ± 3.53	Citrus, floral, fatty	0.21
	Terpineol	22.38	145.36 ± 7.61	Floral, pine wood	2.42
	3,7-Dimethyl-7-octen-1-ol	23.58	8.38 ± 1.32	Floral, fruit, grass	0.42
	cis-3,7-Dimethyl-2,6-octadien-1-ol	23.33	15.98 ± 2.84	Rose, citrus	3.20
	Cis-isogeraniol	24.78	20.47 ± 1.93	Floral, pine wood	2.04
	T-cadinol	24.25	3.95 ± 0.64	-	-
	Beta-acorenol	29.25	5.55 ± 0.24	-	-
	Alpha-cadinol	29.85	1.55 ± 0.11	-	-
	1-Epi-cubenol	29.98	11.11 ± 2.37	Floral, pine wood, citrus	0.37
Ketones	6-Methylhept-5-en-2-one	13.92	1.27 ± 0.33	-	-
	Benzylacetone	24.85	10.16 ± 1.25	Floral, fruit	0.10
Esters	Pinane	19.45	6.84 ± 1.25	Peppermint	0.34
	Ethyl acetate	3.18	7580 ± 109	Fruit, sweets	1.52
	Isoamyl acetate	8.05	469.62 ± 15.74	Banana	0.94
	Isobutyl 2-methylbutyrate	9.72	129.14 ± 8.86	Apple	1.29
	Ethyl hexanoate	11.52	94.6 ± 12.35	Pineapple	4.73
	2-Methyl butyl isovalerate	13.18	1.29 ± 0.31	-	-
	Ethyl caprylate	16.98	196.22 ± 13.95	Fruit, cream	1.96
	Bicyclo [2.2.1] heptan-2-ol, 1,7,7-trimethyl-, 2-acetate	20.32	1.98 ± 0.15	-	-
	Ethyl caprate	21.78	6.07 ± 1.42	-	-
	3,7-Dimethyloct-6-enyl 2-Methylpropanoate	22.05	3.57 ± 0.56	Fruit, rose	0.12
	Neryl acetate	23.11	7.53 ± 1.29	Fruit, citrus	0.75
	3,7-Dimethyl-2,6-octadienyl acetate	23.65	0.54 ± 0.02	-	-
	Isobutyl benzoate	24.05	2.49 ± 0.23	-	-
	Phenethyl acetate	24.32	249.32 ± 15.66	Fruit, rose, honey	4.98
	Ethyl laurate	25.25	15.71 ± 1.04	-	-
	Phenethyl isobutyrate	25.83	5.07 ± 0.23	-	-
	4-Phenyl-2-butyl acetate	25.92	0.88 ± 0.06	-	-
	Phenethyl valerate	26.98	9.71 ± 0.95	-	-
	2-Methylpropyl 3-phenylpropanoate	27.12	6.07 ± 0.62	-	-
	Ethyl palmitate	30.65	14.46 ± 1.07	Fruit, waxy	0.14
	Isobutyl 3-phenylpropanoate	31.08	6.35 ± 0.55	-	-
Acids	Octanoic acid	23.08	9690 ± 209	Fat, cheese, acid	0.97
Ethers	Rose oxide	14.52	0.26 ± 0.01	Fruit, rose	0.26
	2-Methallyl-4-methyl-phenol	17.58	1.12 ± 0.16	Floral, citrus	0.22
	Anethole	24.52	1.81 ± 0.24	-	-

## Data Availability

The original contributions presented in the study are included in the article, further inquiries can be directed to the corresponding author.
